# Simultaneous Videofluoroscopy and Endoscopy for Dysphagia Evaluation in Preterm Infants—A Pilot Study

**DOI:** 10.3389/fped.2020.00537

**Published:** 2020-09-15

**Authors:** Ranjith Kamity, Louisa Ferrara, Vikramaditya Dumpa, Jenny Reynolds, Shahidul Islam, Nazeeh Hanna

**Affiliations:** ^1^Department of Pediatrics, New York University Winthrop Hospital, Mineola, NY, United States; ^2^Department of Pediatrics, New York University Long Island School of Medicine, Mineola, NY, United States; ^3^Department of Communication Sciences and Disorders, Molloy College, Rockville Centre, NY, United States; ^4^Department of Physical Medicine, Baylor University Medical Center, Dallas, TX, United States; ^5^Division of Health Services Research, Department of Foundations of Medicine, New York University Long Island School of Medicine, Mineola, NY, United States

**Keywords:** videofluoroscopy, modified barium swallow study, fiberoptic endoscopic evaluation of swallowing (FEES), dysphagia, swallowing dysfunction, laryngeal penetration, tracheal aspiration, preterm infant

## Abstract

**Introduction:** The assessment of dysphagia in preterm infants has been limited to clinical bedside evaluation followed by videofluoroscopic swallow study (VFSS) in selected patients. Recently, fiberoptic endoscopic evaluation of swallowing (FEES) is being described more in literature for preterm infants. However, it is unclear if one test has a better diagnostic utility than the other in this population. Furthermore, it is also unclear if performing FEES and VFSS simultaneously will increase the sensitivity and specificity of detecting dysphagia compared to either test performed independently.

**Objectives:** The primary objective of this study is to evaluate the feasibility of performing VFSS and FEES simultaneously in preterm infants. Our secondary objective is to determine whether simultaneously performed VFSS–FEES improves the diagnostic ability in detecting dysphagia in preterm infants compared to either test done separately.

**Methods:** In this pilot study, we describe the process involved in performing simultaneous VFSS–FEES in five preterm infants (postmenstrual age ≥36 weeks) with dysphagia. A total of 26 linked VFSS–FEES swallows were analyzed, where the same bolus during the same swallow was compared using simultaneous fluoroscopy and endoscopy. The sensitivity and specificity of detecting penetration and aspiration were evaluated in simultaneous VFSS–FEES compared with each test done independently.

**Results:** Our results demonstrated that performing simultaneous VFSS–FEES is feasible in preterm infants with dysphagia. All patients tolerated the procedures well without any complications. Our pilot study in these five symptomatic preterm infants demonstrated a low incidence of aspiration but a high incidence of penetration. Simultaneous VFSS–FEES (26 linked swallows) improved the ability to detect penetration compared to each test done separately.

**Conclusion:** To our knowledge, this study is the first to demonstrate the feasibility of performing VFSS and FEES simultaneously in symptomatic preterm infants with dysphagia resulting in potentially higher diagnostic yield than either procedure done separately.

## Introduction

Swallowing dysfunction (dysphagia) in preterm infants is significantly under-recognized due to the lack of reliable assessment tools. Videofluoroscopic swallow study (VFSS) is currently the most widely used assessment tool to diagnose dysphagia in preterm infants (1, 2). In recent years, Fiberoptic endoscopic evaluation of swallowing (FEES) has also become a feasible assessment tool in preterm infants (3–6). Although both tests have proven to be valuable and feasible, each test has its benefits and limitations in regard to visualization and assessment of swallowing physiology.

The FEES procedure has several advantages over VFSS (7, 8). The natural feeding environment is not altered with FEES, as it can be performed at the bedside within a more natural feeding environment/position, with the infants' routine feeding liquids as well as during breastfeeding. However, the greatest advantages of FEES are the avoidance of radiation exposure, ease of use, low ongoing cost, the ability to observe the pharyngeal and laryngeal anatomy directly, evaluate pooling secretions in the pharynx/larynx, as well as the ability to repeat the procedure as frequently as indicated. However, FEES does have some limitations, as the laryngeal elevation during swallowing causes the epiglottis to invert, which can temporarily block the view during endoscopy (white-out period), as well as possible discomfort during endoscopy (3, 7). The initial cost of equipment and training necessary to perform FEES is considerable. In addition, all three phases of swallowing (oral, pharyngeal, and esophageal) can be assessed by VFSS, while FEES can only assess the pharyngeal phase.

In adults, VFSS and FEES have a 90% agreement in detecting penetration or aspiration (9). The data agreement in preterm neonates and infants, however, is unclear. The recent FEES study on preterm infants by Suterwala et al. (3) found that the presence and absence of penetration had high agreement using both VFSS (86 and 88%, respectively) and FEES (85 and 72%, respectively). Also, there were high rates of agreement for detecting the absence of aspiration for both VFSS (94%) and FEES (89%). However, low rates of agreement were reported for detecting the presence of aspiration for both VFSS (43%) and FEES (0%).

The knowledge that early feeding skills in preterm infants can vary greatly from feeding to feeding, or even across a given feeding (10), presents a limitation to all the prior agreement data as each test was performed separately at different time points. A true measure of agreement cannot be established unless both procedures are performed simultaneously during the same swallow. We hypothesize that performing VFSS and FEES simultaneously (VFSS–FEES) is feasible in preterm infants and will improve the sensitivity and specificity of detecting swallowing dysfunction in preterm infants compared with each test performed independently. To the best of our knowledge, this study is the first to demonstrate the feasibility of performing VFSS and FEES simultaneously during the same feeding in preterm infants with dysphagia, where the same bolus and the same swallow were compared on “linked swallows.” In this paper, our primary objective was to describe the methodology of performing simultaneous VFSS-FEES in preterm infants presenting with dysphagia. Our secondary objective was to determine whether simultaneously performed VFSS–FEES would have a diagnostic advantage in detecting dysphagia in preterm infants compared to either test done separately.

## Materials and Methods

### Setting

We describe a pilot study of five preterm infants who underwent VFSS and FEES simultaneously for dysphagia evaluation between January 1, 2017, and December 31, 2018, at NYU Winthrop Neonatal Intensive Care Unit. All infants followed our standard clinical protocol, which involved referral by the medical team to the speech and language pathologist (SLP) to evaluate possible dysphagia further. The SLP performed a clinical feeding and swallowing evaluation and then determined if the infants required further diagnostic imaging assessment (VFSS, FEES, or VFSS–FEES). After discussion with the infants' medical team, patients over 35 weeks postmenstrual age, weighing over 2 kg, maintaining stable temperature in open crib, and on minimal (nasal cannula ≤1 L/min) or no respiratory support were selected for the combined VFSS–FEES procedure if dysphagia was perceived to be significant clinically.

Demographics were collected, including gestational age at birth, chronological age, gender, birth weight, and type of respiratory support at the time of the study. The presence or absence of laryngeal penetration and tracheal aspiration was assessed on VFSS and FEES performed simultaneously. Laryngeal penetration (penetration) was defined as the presence of liquid within the laryngeal vestibule on or above the true vocal folds, and tracheal aspiration (aspiration) was defined as the occurrence of liquid below the level of the true vocal folds during individual swallows (11). Data collection was approved by NYU School of Medicine's Institutional Review Board in accordance with institutional policies.

### Preparation

The procedures were performed in the fluoroscopy suite in the Department of Radiology. The team included the neonatologist, SLP, pediatric radiologist, infant's bedside registered nurse, and pediatric otorhinolaryngologist. The feeding therapy team set up the endoscopy unit within the radiology suite prior to the procedure. The infants were transported to the radiology suite in a heated isolette.

Thin barium (Varibar, Bracco Diagnostics, Monroe Township, NJ, USA) was prepared with 50% dilution as per Fink and Ross (12). A total of 30 ml of the mixed barium liquid was poured into a Similac volu-feeder (Abbott Nutrition, Lake Forest, IL, USA). Two drops of McCormick Green Food Color (Sparks, MD, USA) were added to the bottle and stirred until evenly distributed to enable clear visualization of the bolus. A Similac slow-flow nipple (Abbott Nutrition, Lake Forest, IL, USA) was used for the initial trial with thin barium. Barium at different thicknesses (nectar and honey-thick) and nipples of various flow rates were set up in advance as per our standard practice.

### Procedure for Simultaneous Videofluoroscopic Swallow Study–Fiberoptic Endoscopic Evaluation of Swallowing

**Figure 1:**
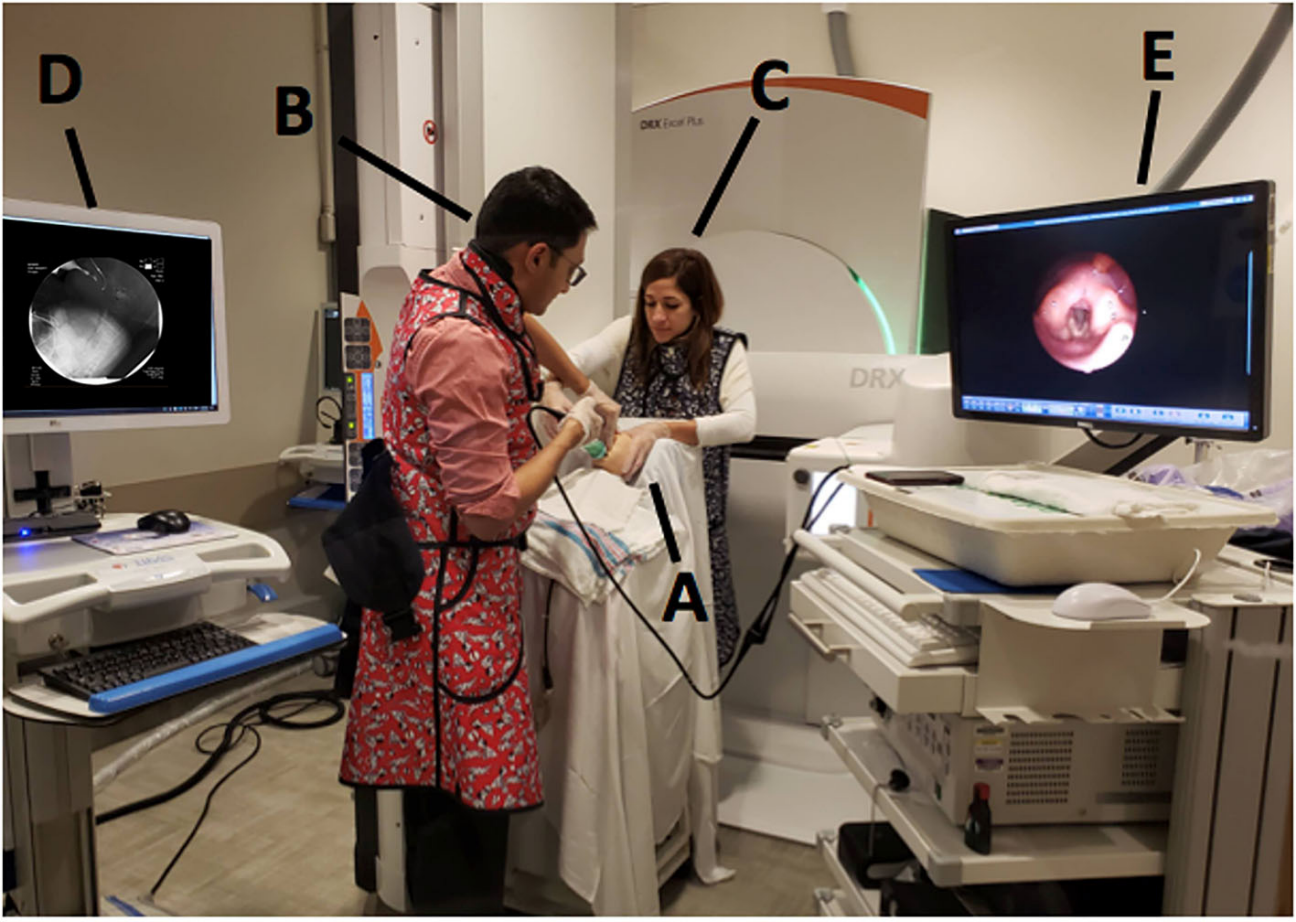
Mock videofluoroscopic swallow study (VFSS)–fiberoptic endoscopic evaluation of swallowing (FEES) procedure with simulated images. **(A)** Infant; **(B)** Endoscopist; **(C)** Feeder; **(D)** VFSS monitor; **(E)** FEES monitor.

In order to perform both tests simultaneously, each team member had a specific assigned task and location (Figure 1). The endoscopist stood immediately in front of the infant with the FEES monitor to the right of their vision. The feeder stood behind the infant with the VFSS monitor positioned in a direct line of vision, just above or to the side of the endoscopist. The rest of the feeding team surrounded the VFSS machine to assist as needed. All personnel employed appropriate radiation safety measures using lead shields.

The infants were tightly swaddled with hands at the midline. The infants were placed in a semi-reclined position with a 45°-90° angle in a Tumble Form Infant Seat (J.A. Preston, Jackson, MI, USA) attached to a MAMA System (MAMA Systems, Inc., Oconomowoc, WI, USA). Participants were protected against radiation exposure by the placement of a small lead shield over their pelvic area. A pulse oximeter (Masimo Corporation, Irvine, CA, USA) remained in use during the transport and for the entirety of the evaluation to record heart rate and percent oxygen saturation.

**Figure 2:**
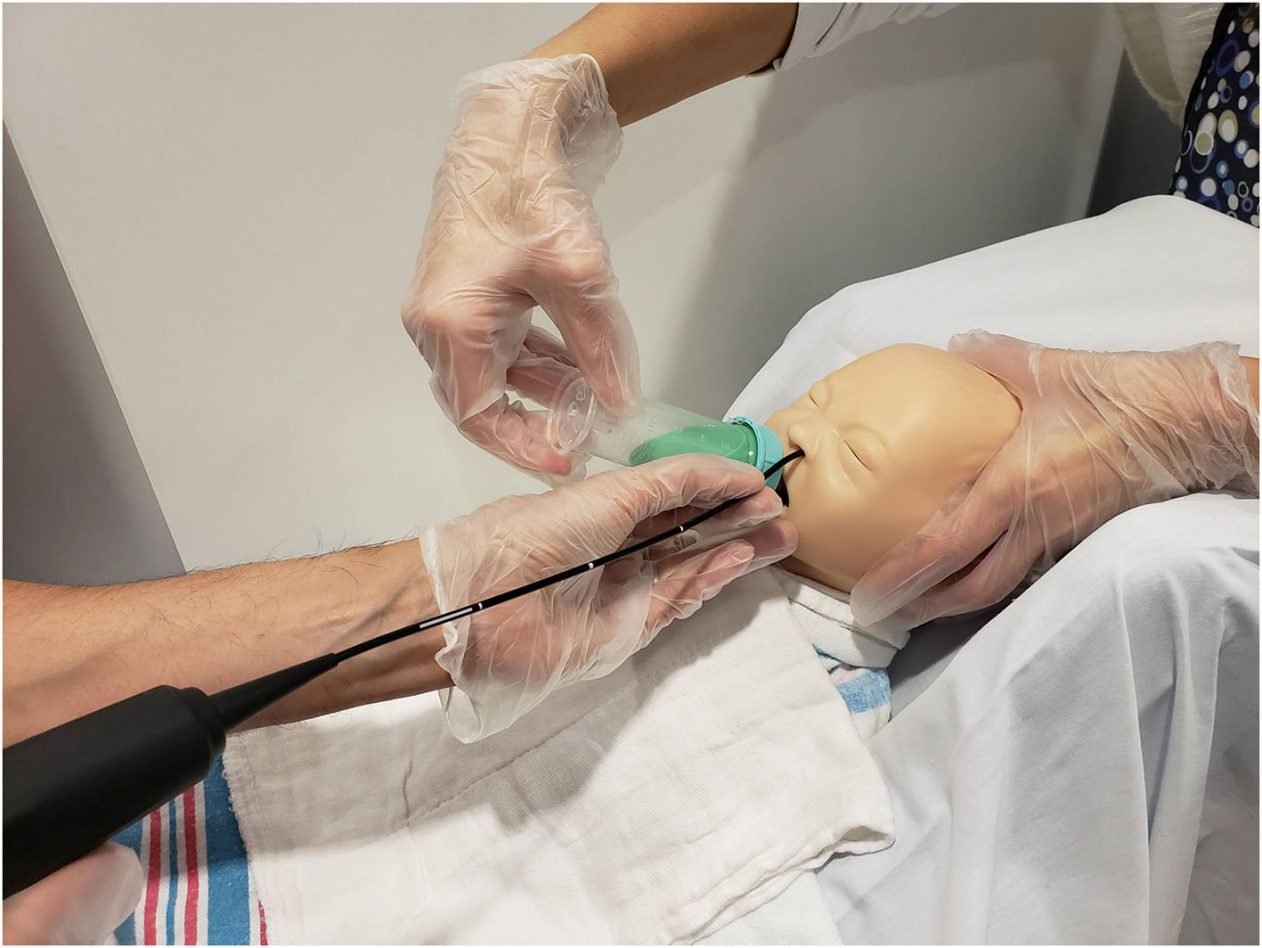
Mock videofluoroscopic swallow study (VFSS)–fiberoptic endoscopic evaluation of swallowing (FEES) procedure. Note that the endoscopist is inserting the FEES scope while the feeder stabilizes the infant's head and holds the bottle. To avoid radiation exposure and to minimize interference with VFSS, the feeder's arm is arched and the endoscopist stabilizes the FEES scope against the infant's chin. Radiologist employs tight coning to limit radiation field further.

**Figure 3:**
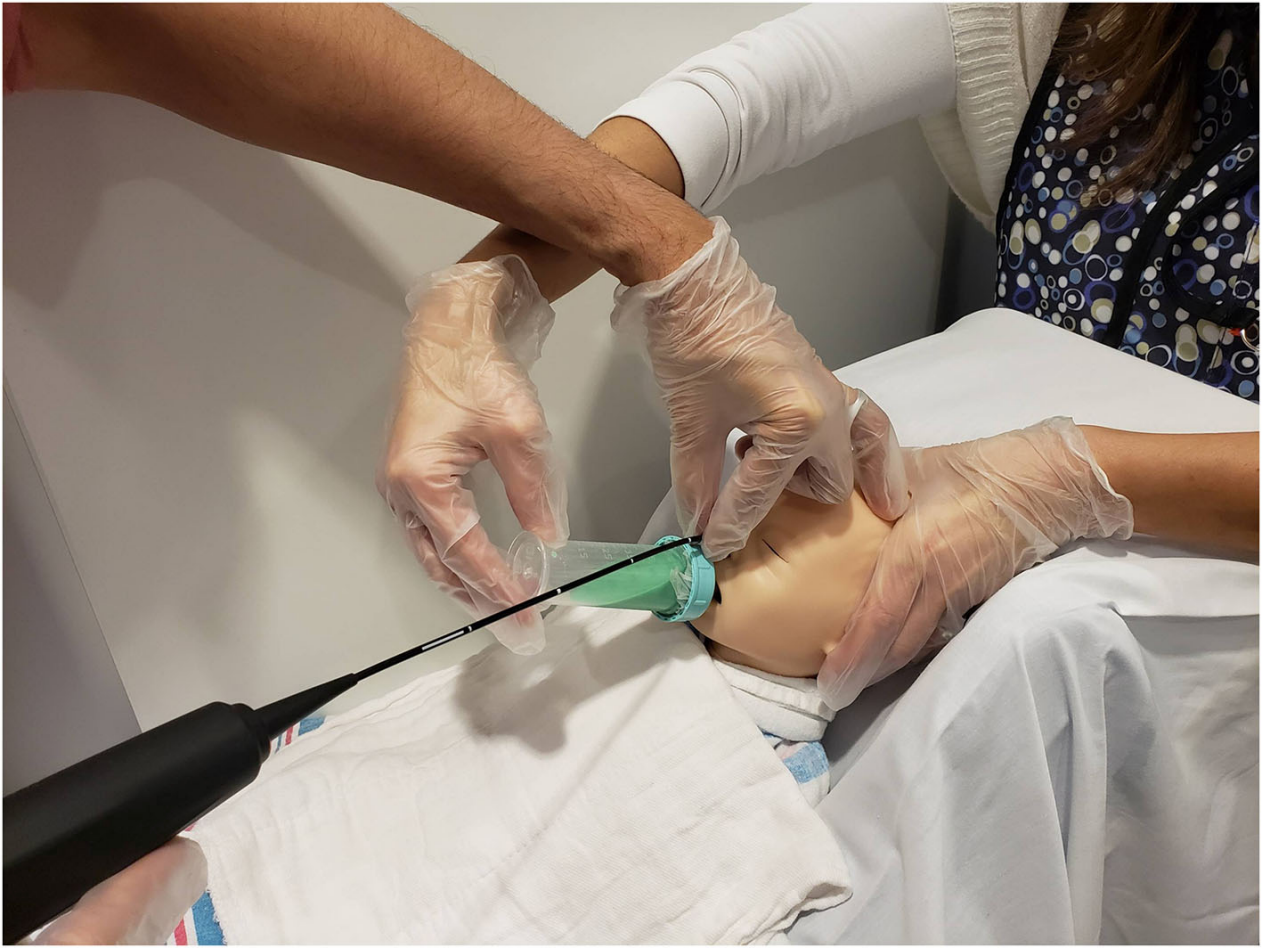
Mock videofluoroscopic swallow study (VFSS)–fiberoptic endoscopic evaluation of swallowing (FEES) procedure. Image offered to demonstrate an alternative way to hold the FEES scope. The endoscopist arches his/her hand and anchors on the forehead while moving the hand out of the VFSS view while the feeder's arm is also arched. This hold was performed on infants who moved their heads more often during the procedure because the endoscopist's hand placement on the infant's forehead helped reduce infant movement.

The pediatric radiologist positioned the camera head of the videofluoroscopy machine to obtain a lateral view and narrowed the field of view via coning to reduce radiation exposure. Once the infant was appropriately positioned, the FEES endoscopist passed the fiberscope trans-nasally and guided the scope through the nasal cavity into the pharynx for a high position (13) while the feeder stabilized the infants' head. No topical anesthesia or decongestant was used (3, 7). During scope placement, the feeder kept the infant calm with a pacifier and, if required, using a 24% sucrose solution (Sweet-Ease Natural, Philips Mother and Child Care, Koninklijke Philips NV, USA) for 2 min prior to and during insertion. The feeder and endoscopist modified their hand position to avoid getting in the path of radiation and to minimize interference with the VFSS view (Figure 2). Figure 3 shows an alternative hold used by the endoscopist and the feeder in order to stabilize the FEES scope in relation to the infant's face.

The anatomy of laryngeal and pharyngeal structures was first visualized on FEES. The feeder then substituted the pacifier for the bottle. Once the infant organized to a nutritive sucking pattern, the VFSS was turned on only during the time of the barium swallows to limit radiation exposure. Fluoroscopy was set to 30 frames per second (fps). The pediatric radiologist assisted in the assessment of anatomy and swallowing physiology, as viewed on fluoroscopy. The pacing technique was used if the infant demonstrated suck, swallow, breathe incoordination, or significant signs of stress. Each swallow identified on VFSS was numbered, and any signs of dysfunction identified on the VFSS were called out loud. This assisted in time-stamping each swallow for FEES and VFSS image comparisons, as both the recording equipment had audio recording capabilities. The identification of significant swallowing deficits on VFSS, assessed in real time, dictated the advancement of trials, including liquid thickness and nipple flow rate modifications.

To evaluate the safety of the infants, their vital signs, including heart rate and oxygen saturation, were monitored using pulse oximetry. The presence of any adverse reactions, such as epistaxis, vasovagal response, laryngospasm, respiratory distress, apnea, cyanosis, tachycardia, and bradycardia, was recorded. The neonatologist was prepared for resuscitation, as was standard with all FEES procedures in our unit.

### Image Capture

Fluoroscopic images (VFSS) were captured using Philips' Easy Diagnostic digital fluoro-radiographic unit (Philips Healthcare USA) and simultaneously recorded and saved electronically onto the TIMS DICOM System (Foresight Imaging, Chelmsford, MA, USA). Endoscopic images (FEES) were captured using Pentax 2.4-mm fiber-scope and simultaneously recorded and saved electronically onto Digital Swallowing Workstation (Pentax Medical, Montvale, NJ, USA).

### Data Collection and Analysis

**Figure 4:**
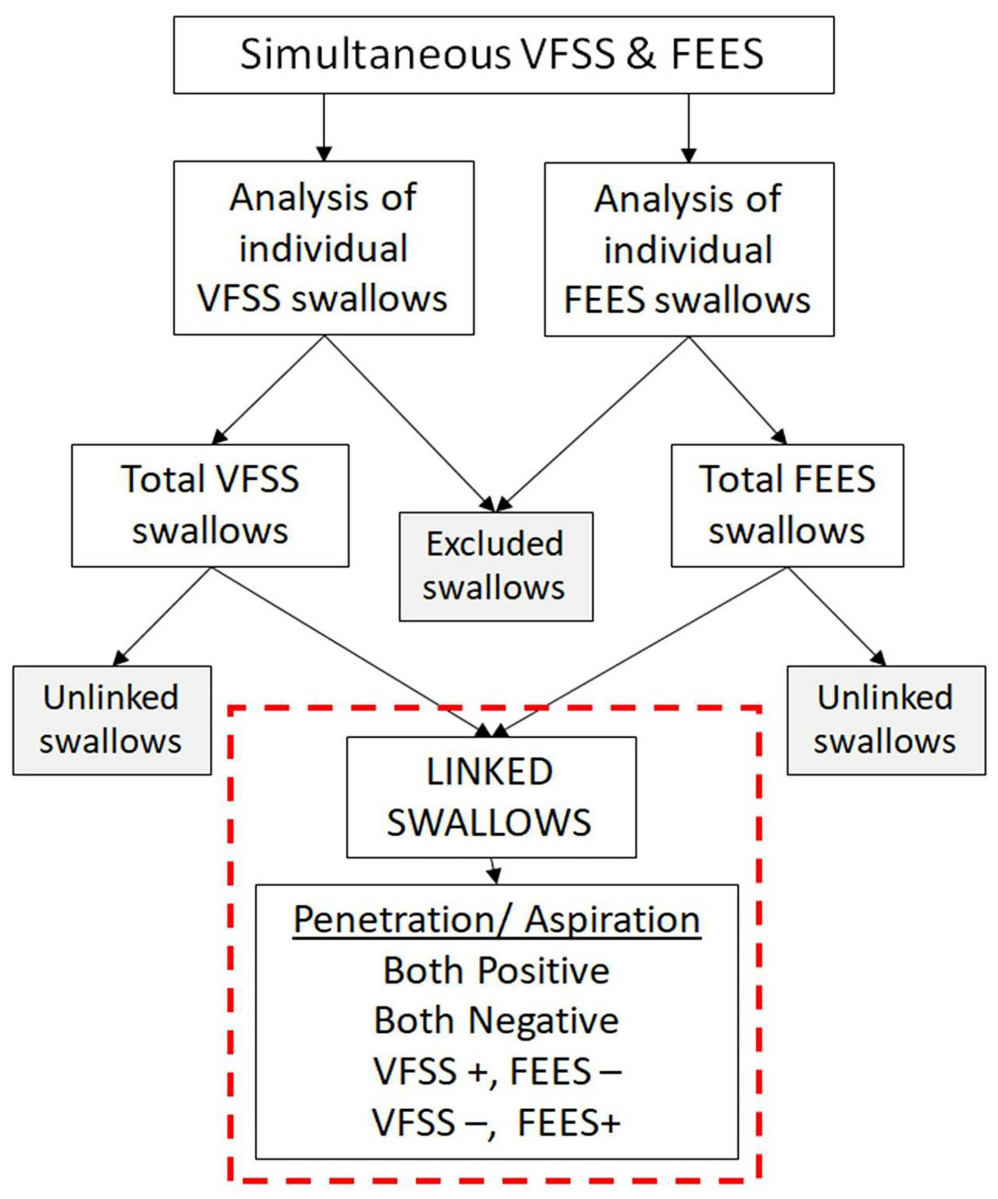
Overview of swallow analysis. All the swallows from videofluoroscopic swallow study (VFSS) and fiberoptic endoscopic evaluation of swallowing (FEES) procedures were reviewed individually for penetration and aspiration. Swallows not meeting our image quality criteria were excluded. “Linked swallows” include swallows comparing the same bolus during the same swallow on VFSS and FEES simultaneously.

**Figure 5:**
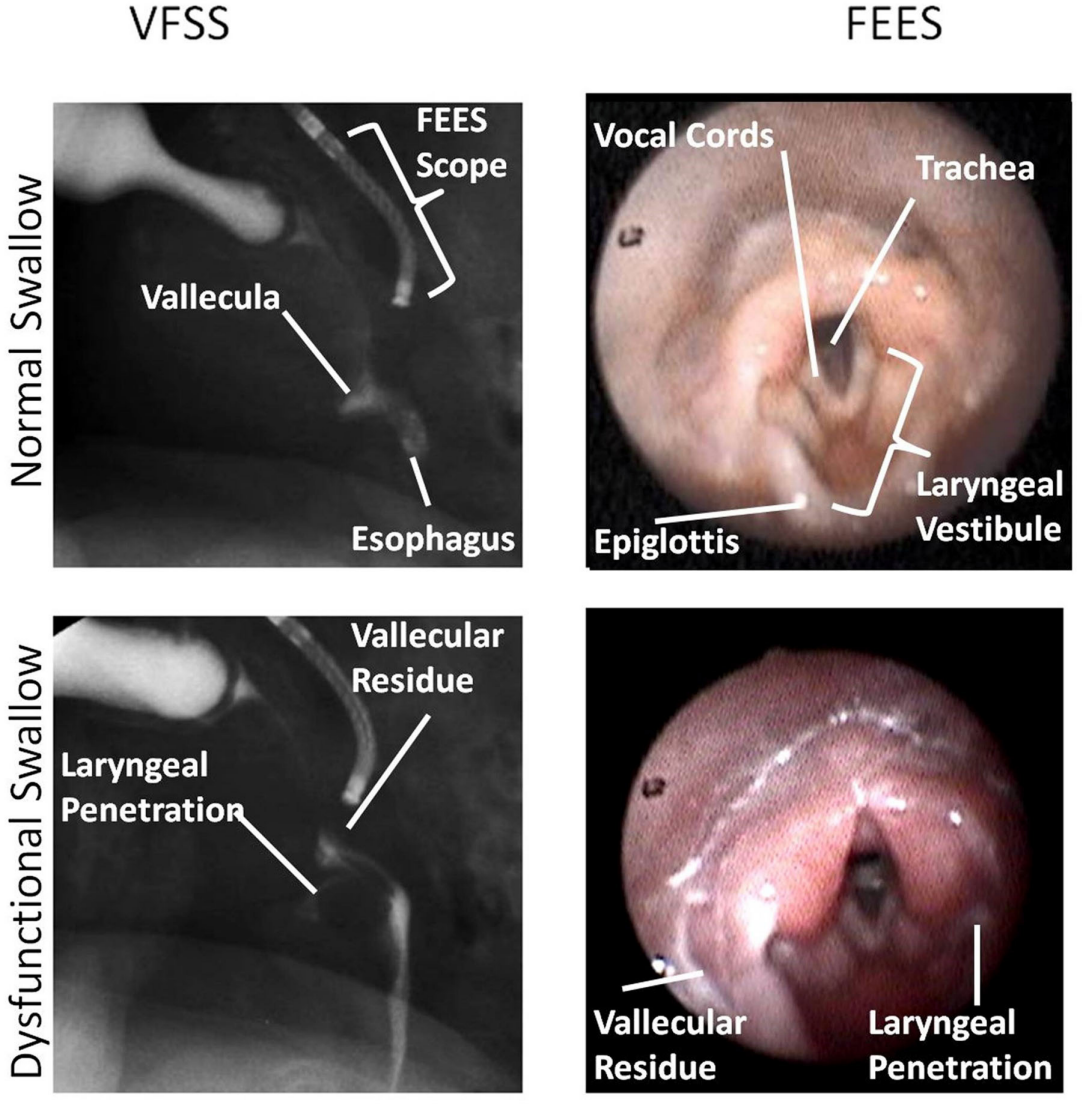
This figure shows an identical swallow on videofluoroscopic swallow study (VFSS) and fiberoptic endoscopic evaluation of swallowing (FEES) still-frame images for subject 1. In this image, FEES images are two frames after VFSS images once the white-out period is over. Anatomic landmarks are identified as noted. Laryngeal penetration could be visualized on both VFSS and FEES. Images were obtained with parental consent.

Swallows captured on both the studies (VFSS and FEES) during the simultaneous VFSS–FEES procedure were analyzed independently for penetration and aspiration. Individual FEES swallows had to meet our image quality criteria to be included for analysis, which included a clear image with no obstruction to view that could potentially alter its interpretation. Figure 4 shows an overview of swallow analysis. We then identified “linked swallows” which were captured on both VFSS and FEES simultaneously. An example of simultaneous endoscopic and fluoroscopic views of a linked swallow is shown in Figure 5. Each linked swallow was analyzed independently for the presence or absence of penetration or aspiration within the FEES and VFSS images. We sought agreement between the linked swallows on VFSS and FEES by labeling each swallow with one of four classifications as follows:

1. Agreement–Positive on both VFSS and FEES
2. Agreement–Negative on both VFSS and FEES
3. Disagreement–FEES Positive and VFSS Negative
4. Disagreement–VFSS Positive and FEES Negative.

To increase the reliability of our findings, two reviewers independently identified penetration and aspiration on each of the identified linked swallows using time-marked data and available audio recording on FEES and VFSS (TIMS). The swallows with similar findings, as assessed by the two reviewers, were included automatically. The swallows with different findings between the two reviewers were reassessed by them together to arrive at a consensus. A third reviewer was used as a tiebreaker if an agreement was not reached. If still unable to reach a conclusion, the swallows were excluded from the study. However, this did not occur in any of the linked swallows analyzed.

The results of combined VFSS and FEES were compared to each test alone for penetration and aspiration. For the simultaneous VFSS–FEES measure, similar to Giraldo-Cadavid et al. (14) and Armstrong et al. (4) we used a composite measure for diagnosing penetration or aspiration if they were present on either VFSS “OR” FEES. If penetration or aspiration was found on either procedure, it was considered a “positive” finding, while a “negative” finding would require both tests to be negative.

## Statistical Methods

This is a pilot study with five unique patients with 26 linked swallows and a paired design for linked swallow analysis. Each linked swallow was assessed using VFSS and FEES procedures performed simultaneously. Each swallow served as its own control for the other procedure.

Two methods were used to measure agreement between VFSS and FEES for each classification (penetration and aspiration) on linked swallows: Exact McNemar's test of agreement and Cohen's kappa (κ) coefficient. Percent agreement was calculated as the ratio of the number of times two tests agreed on each swallow divided by the total number of swallows analyzed. The kappa statistic estimates the proportion of agreement among tests after removing the proportion of agreement that would occur by chance. Separate κ coefficients and corresponding 95% confidence intervals were calculated for the agreement among the tests. The following guidelines outlined by Landis and Koch (15) will be used to characterize kappa values: Poor agreement (<0), slight agreement (0.0–0.20), fair agreement (0.21–0.40), moderate agreement (0.41–0.60), substantial agreement (0.61–0.80), and almost perfect agreement (0.81–1.00).

Sensitivity, specificity, positive predictive value (PPV), and negative predictive value (NPV), along with their corresponding 95% confidence intervals, were calculated to measure the accuracy of linked swallows on FEES alone in separately assessing penetration and aspiration with respect to the VFSS (FEES vs. VFSS; VFSS vs. FEES). Since there is no established gold standard, we compared VFSS and FEES, assuming each procedure as the gold standard against the other. The same diagnostic testing analyses were performed to measure the accuracy of VFSS and FEES with respect to the composite VFSS–FEES measure as the gold standard (FEES vs. VFSS-FEES and VFSS vs. VFSS-FEES).

Sensitivity and specificity were also calculated to compare the complete individual patient studies to each other (FEES and VFSS). In addition, the percentages of penetration and aspiration identified on all swallows (linked and unlinked) were compared between VFSS and FEES via a two-sample test of proportion. A result was considered statistically significant at the *p* < 0.05 level of significance. As a pilot study is limited in terms of statistical power due to its small sample size, no inferential decision was made based on p-values. All analyses were performed using SAS version 9.4 (SAS Institute, Cary, NC).

## Results

We report the results from five patients, as described in Table 1. All infants were over 35 weeks' postmenstrual age and weighing over 2 kg at the time of the study. All five infants were born premature between 23.9 and 35.3 weeks' gestation. Three of the five infants had bronchopulmonary dysplasia, of which two were on nasal canula (<1 L/min) at the time of the simultaneous procedure. None of the infants included had major comorbidities, including anatomic defects, especially related to the upper airway. All patients received feeding modifications after the procedures and eventually were discharged home feeding orally. The average time to discharge was 8.4 days after undergoing the procedures. All the infants tolerated the simultaneous procedures well with no change in vital signs or any noted complications before, during, or after the procedure.

**Table 1:** Demographic characteristics of infants who underwent simultaneous VFSS–FEES.

	**Gestational age (weeks)**	**Corrected gestational age (weeks)**	**Sex**	**Birth weight (g)**	**Weight (g) at study**	**RESP support**	**Comorbidities**	**Adverse events**
1	23.9	41.7	M	607	3,665	NC 1L	RDS, BPD	None
2	33.6	37.7	F	1,760	2,520	RA	RDS, GER	None
3	35.3	36.7	M	2,815	2,615	RA	RDS	None
4	30.3	39.4	M	930	2,380	NC 1L	RDS, BPD	None
5	30.0	39.6	M	990	2,646	RA	RDS, BPD Twin	None

*Adverse events include nasal bleeding, vasovagal response, laryngospasm, respiratory distress, apnea, cyanosis, tachycardia, and bradycardia. VFSS, videofluoroscopic swallow study; FEES, fiberoptic endoscopic evaluation of swallowing; BPD, bronchopulmonary dysplasia; GER, gastroesophageal reflux; RDS, respiratory distress syndrome; NC, nasal canula; RA, room air*.

### Analysis of Linked Swallows on Simultaneous Videofluoroscopic Swallow Study–Fiberoptic Endoscopic Evaluation of Swallowing

A total of 66 linked swallows were captured, with 26 linked swallows meeting our image quality criteria for simultaneous analysis. No aspiration was detected in any of these 26 linked swallows. For penetration, the analysis of linked swallows showed 73% agreement (19 swallows; both negative or both positive for penetration) and a 27% disagreement between VFSS and FEES findings for simultaneous analysis (Table 2). The two reviewers agreed on the findings for 100% of FEES swallows (26/26) and 92% VFSS images (24/26), needing a third reviewer as a tiebreaker. Table 3 shows a comparative analysis of linked swallows showing sensitivity, specificity, positive and negative predictive values of VFSS, FEES, and composite VFSS–FEES with one another.

**Table 2:** Agreement between videofluoroscopic swallow study (VFSS) and fiberoptic endoscopic evaluation of swallowing (FEES) for penetration identified on linked swallows.

		**Penetration *n* (%)**
1	VFSS and FEES+	11 (42%)
2	VFSS and FEES–	8 (31%)
3	VFSS+/FEES–	2 (8%)
4	VFSS–/FEES+	5 (19%)
	TOTAL AGREEMENT (1+2) *n* (%)	19 (73%)
	TOTAL DISAGREEMENT (3+4) *n* (%)	7 (27%)
	KAPPA *k* (*p*)	0.46 (0.453)

*The total agreement was 73% (19/26), with a kappa of 0.46 (moderate agreement). No aspiration was identified on linked swallows*.

**Table 3:** Comparisons for linked swallows.

**Study being tested**	**Presumed gold standard**	**Sensitivity**	**Specificity**	**PPV**	**NPV**	**Kappa (95% CI)**	***p*-value^†^**
FEES	VFSS	85	62	69	80	0.46 (0.13–0.79)	0.453
VFSS	FEES	69	80	85	62		
VFSS	VFSS–FEES	72	100	100	62	0.62 (0.34–0.90)	0.063
FEES	VFSS–FEES	89	100	100	80	0.83 (0.61–1.00)	0.5

*This table depicts the comparison of individually linked swallows captured on both fiberoptic endoscopic evaluation of swallowing (FEES) and videofluoroscopic swallow study (VFSS). A composite outcome was used for simultaneous VFSS–FEES (Positive, positive on either VFSS or FEES; Negative, negative on both VFSS and FEES). PPV, positive predictive value; NPV, negative predictive value*.

† *P-values are from Exact McNemar's test, and a value >0.05 suggests there is insufficient evidence of disagreement between swallow studies*.

### Analysis of All Swallows (Including All Linked and Unlinked Swallows)

Using each swallow as an independent measure unit, a total of 216 swallows were assessed on VFSS compared to 107 on FEES, including linked and unlinked swallows (Table 4). FEES identified laryngeal penetration on 55.1% of all swallows compared to VFSS at 23.6% (*p* < 0.01). FEES identified tracheal aspiration in 3.7% of all swallows vs. 8.3% with VFSS (*p* = 0.12).

**Table 4:** Videofluoroscopic swallow study (VFSS) vs. fiberoptic endoscopic evaluation of swallowing (FEES) for all swallows (linked and unlinked).

	**Positive**	**Total**	**%**
VFSS aspiration	18	216	8.3%
FEES aspiration	4	107	3.7%
VFSS penetration	51	216	23.6%
FEES penetration	59	107	55.1%

*This table shows the results of all swallows on VFSS and FEES. FEES identified penetration significantly more than VFSS (p < 0.001), while there was no significant difference between VFSS and FEES for aspiration (p = 0.123)*.

### Study Comparison: Videofluoroscopic Swallow Study vs. Fiberoptic Endoscopic Evaluation of Swallowing for All Infants

Analysis of the complete studies using each patient as an independent measure unit showed that all five infants (100%) had penetration (defined as any swallow during the procedure showing penetration) on both studies. For aspiration (defined as any swallow during the procedure showing aspiration), FEES was positive in three patients, while VFSS was positive for four out of five patients (Table 5). In this limited number of patients, using VFSS as the gold standard, FEES had a sensitivity of 75% for aspiration and 100% for penetration with a specificity of 100% for aspiration.

**Table 5:** Videofluoroscopic swallow study (VFSS) and fiberoptic endoscopic evaluation of swallowing (FEES) complete study results for five pilot patients.

**Penetration**	**VFSS**		
FEES		Positive	Negative
	Positive	5	0
	Negative	0	0
**Aspiration**	**VFSS**		
FEES		Positive	Negative
	Positive	3	0
	Negative	1	1

*In this table, FEES is compared to the current gold standard VFSS. FEES had a sensitivity of 75% for aspiration and 100% for penetration with a specificity of 100% for aspiration*.

## Discussion

To our knowledge, our study is the first to report the methodology, feasibility, and safety of performing simultaneous VFSS and FEES in preterm infants suspected of having dysphagia. The procedure was well-tolerated, with no adverse events noted. Our results are significant, first, because it establishes a novel methodology aimed at improving test sensitivity and specificity for dysphagia diagnosis in preterm infants, and second, it sets the stage for using such procedures in future studies to compare the diagnostic utility of VFSS vs. FEES in various clinical settings in preterm infants with dysphagia.

Dysphagia in preterm infants represents a major challenge for health care providers, given the potential health risks associated with such disease in this vulnerable population. It is estimated that 30–70% of very low birth weight preterm infants (birth weight <1,500 g) will be diagnosed with dysphagia (16–18). Repeated aspiration can result in a persistent inflammatory state and chronic lung injury that can be devastating for already fragile and developmentally immature lungs in preterm infants. There is a substantial lack of dysphagia research concerning preterm infants, which has hindered any evidence-based approach to diagnosing and treating this common problem. One of the reasons is the lack of practical assessment tools to diagnose dysphagia in preterm infants.

Until recently, VFSS was the only available tool to study dysphagia in preterm infants (1, 2). This procedure has several disadvantages (1, 19), including the use of radiation, the inability for use during breastfeeding, and the requirement that the infant must be transported to a radiology suite (7), which can be risky for sick preterm infants. The introduction of FEES, with its various advantages over VFSS, set the stage for a new era in dysphagia diagnosis in preterm infants. In adults, the discussion continues regarding which test should be the gold standard for dysphagia diagnosis. A similar dilemma exists in pediatric patients. Simultaneous VFSS–FEES procedures done in adults helped to identify the advantages and disadvantages of each procedure performed in different clinical settings (20, 21). Our study shows that performing simultaneous VFSS–FEES is feasible in preterm infants and is well-tolerated.

Our secondary objective was to determine whether simultaneously performed VFSS–FEES would have a diagnostic advantage in detecting dysphagia in preterm infants compared to either test done separately. Although the study was not powered for this outcome, our results showed that simultaneously performed VFSS–FEES had higher diagnostic value for penetration diagnosis compared to each test done separately. Literature shows that penetration is linked to tracheal aspiration (22). Our pilot data showed that FEES detected more penetration than VFSS, which corroborates with available evidence reported in both the pediatric (4, 23–25) and adult literature (20, 26, 27). This supports the notion that FEES is more sensitive than VFSS in detecting penetration. In our linked swallows, there were no aspiration episodes but a significant prevalence of penetration. We believe that future studies with more patients comparing more linked swallows are necessary to arrive at a definitive conclusion regarding the diagnostic utility of aspiration events using simultaneous VFSS–FEES.

A recent study by Armstrong et al. (4) compared nonsimultaneous VFSS and FEES (performed at different time points) in preterm infants showing a high agreement between VFSS and FEES for detecting aspiration (92%) and moderate agreement for penetration (56%). Our study showed a 73% agreement between the two procedures for penetration on linked swallows. Armstrong et al. (4) also showed that FEES detected more instances of penetration, which was consistent with our results in this study. The analysis of total swallows captured on VFSS and FEES showed similar results to the linked swallows, implying that the linked swallows accurately represented the whole sample of swallows captured.

Despite the diagnostic advantages of simultaneous VFSS–FEES, performing these two procedures simultaneously may not be relevant for all infants requiring evaluation for dysphagia, as it is best to limit radiation exposure during VFSS whenever possible. However, in questionable or challenging cases, there may be a need to have a highly sensitive and specific test such as simultaneous VFSS–FEES. In our experience with this population, the epiglottis blocked the view during consecutive swallows on several images obtained during FEES (white-out) (7). In comparison, the VFSS images showed all phases of swallowing more consistently. There were instances when visualization of the laryngeal vestibule and airway protection was partially obstructed or missed during the FEES image, but the VFSS image clearly identified aspiration or penetration. Without performing both assessments simultaneously, we would have missed some episodes of penetration or aspiration in those infants. Further studies are needed to confirm the utility and the diagnostic advantage of performing simultaneous VFSS–FEES.

Although our pilot study shows the potential benefits of using simultaneous VFSS–FEES to identify infant dysphagia, this technique is not without limitations. First, the logistics of coordinating all required personnel, equipment maintenance, and availability of the radiology suite required advanced planning, which may be challenging for many institutions. Although this was initially time-consuming and complicated to coordinate, we assume both will be reduced once the combined procedure becomes routine practice. Secondly, we are not aware of any available software programs to synchronize VFSS with FEES images. Therefore, the comparisons had to be performed using two separate computers, running two different programs (TIMS for VFSS, and Digital Swallowing Workstation for FEES), which made the task of identifying and comparing linked swallows tedious and time-consuming.

Another limitation of our study was the subjects' selection bias. Infants in our study had more significant dysphagia since the testing was performed based on clinical necessity and at the discretion of the attending neonatologist. However, this is relevant in clinical practice since symptomatic infants generally tend to receive dysphagia evaluation. However, this procedure combined two separate procedures to be done simultaneously, thereby avoiding two separate procedures on these patients. Hence, the direct costs of performing the procedures simultaneously would be no different from performing both procedures simultaneously. Despite the limitations, this study reports a detailed methodology with the feasibility and safety of simultaneously performed VFSS and FEES in this unique patient population.

## Conclusion

Our study showed that performing VFSS–FEES simultaneously is technically feasible, safe, and will improve the sensitivity and specificity of dysphagia diagnosis compared with each assessment done independently. These results also set the stage for using such procedure in future clinical trials to compare the efficacy and validity of VFSS vs. FEES in various clinical settings as well as guide management strategies, such as nipple flow rate (28), pacing technique (29), and liquid modifications (30, 31) to improve dysphagia symptoms in preterm infants. The simultaneous VFSS–FEES procedure has the potential to change the paradigm of how the diagnosis and treatment of dysphagia are approached for preterm infants and provide clinicians with a novel technique that can impact clinical practice in neonatal intensive care units.

## Data Availability Statement

The datasets generated for this study are available on request to the corresponding author.

## Ethics Statement

The studies involving human participants were reviewed and approved by NYU School of Medicine Institutional Review Board. Written informed consent from the participants' legal guardian/next of kin was not required to participate in this study in accordance with the national legislation and the institutional requirements.

## Author Contributions

NH, RK, VD, and LF were involved in study design, performing the procedures, data collection, and review and analysis. JR was involved in data review and analysis. SI was involved in performing statistical analysis. RK wrote the first draft of the manuscript. All authors critically reviewed, revised, and approved the manuscript in its final version.

## Conflict of Interest

The authors declare that the research was conducted in the absence of any commercial or financial relationships that could be construed as a potential conflict of interest.
